# Fueling the flames of colon cancer – does CRP play a direct pro-inflammatory role?

**DOI:** 10.3389/fimmu.2023.1170443

**Published:** 2023-03-17

**Authors:** Anne Helene Køstner, Anniken Jørlo Fuglestad, Jeanette Baehr Georgsen, Patricia Switten Nielsen, Kristina Bang Christensen, Helle Zibrandtsen, Erik Thorlund Parner, Ibraheem M. Rajab, Lawrence A. Potempa, Torben Steiniche, Christian Kersten

**Affiliations:** ^1^ Center for Cancer Treatment, Sorlandet Hospital, Kristiansand, Norway; ^2^ Department of Clinical Science, University of Bergen, Bergen, Norway; ^3^ Department of Oncology, Akershus University Hospital, Nordbyhagen, Norway; ^4^ Department of Pathology, Aarhus University Hospital, Aarhus, Denmark; ^5^ Department of Clinical Medicine, Aarhus University, Aarhus, Denmark; ^6^ Section for Biostatistics, Department of Public Health, Aarhus University, Aarhus, Denmark; ^7^ College of Science, Health and Pharmacy, Roosevelt University Schaumburg, Schaumburg, IL, United States

**Keywords:** systemic inflammation, C-reactive protein (CRP), CRP isoforms, monomeric CRP, colon cancer, immunohistochemistry (IHC), biomarkers, tumor microenvironment

## Abstract

**Background:**

Systemic inflammation, diagnostically ascribed by measuring serum levels of the acute phase reactant C-reactive protein (CRP), has consistently been correlated with poor outcomes across cancer types. CRP exists in two structurally and functionally distinct isoforms, circulating pentameric CRP (pCRP) and the highly pro-inflammatory monomeric isoform (mCRP). The aim of this pilot study was to map the pattern of mCRP distribution in a previously immunologically well-defined colon cancer (CC) cohort and explore possible functional roles of mCRP within the tumor microenvironment (TME).

**Methods:**

Formalin-fixed, paraffin-embedded (FFPE) tissue samples from 43 stage II and III CC patients, including 20 patients with serum CRP 0-1 mg/L and 23 patients with serum CRP >30 mg/L were immunohistochemically (IHC) stained with a conformation-specific mCRP antibody and selected immune and stromal markers. A digital analysis algorithm was developed for evaluating mCRP distribution within the primary tumors and adjacent normal colon mucosa.

**Results:**

mCRP was abundantly present within tumors from patients with high serum CRP (>30 mg/L) diagnostically interpreted as being systemically inflamed, whereas patients with CRP 0-1 mg/L exhibited only modest mCRP positivity (median mCRP per area 5.07‰ (95%CI:1.32-6.85) vs. 0.02‰ (95%CI:0.01-0.04), p<0.001). Similarly, tissue-expressed mCRP correlated strongly with circulating pCRP (Spearman correlation 0.81, p<0.001). Importantly, mCRP was detected exclusively within tumors, whereas adjacent normal colon mucosa showed no mCRP expression. Double IHC staining revealed colocalization of mCRP with endothelial cells and neutrophils. Intriguingly, some tumor cells also colocalized with mCRP, suggesting a direct interaction or mCRP expression by the tumor itself.

**Conclusion:**

Our data show that the pro-inflammatory mCRP isoform is expressed in the TME of CC, primarily in patients with high systemic pCRP values. This strengthens the hypothesis that CRP might not only be an inflammatory marker but also an active mediator within tumors.

## Background

Systemic inflammation, diagnostically ascribed by measuring levels of the acute phase protein CRP in serum, has consistently been correlated with poor outcomes across cancer types (1–3). However, the biological relationship between CRP and inflammation remained unresolved and controversial for decades. Recently, evidence has been advanced showing that CRP exists in different structural isoforms with distinct biological activities (4). The circulating CRP isoform is a highly soluble pentameric molecule (pCRP) composed of 5 identical globular subunits arranged in a ring-shaped structure (5). Each subunit contains a calcium dependent binding site enabling interaction with phosphocholine (PC), a major component of plasma membranes, defined as the primary ligand for pCRP. However, for the PC ligand to become accessible for CRP binding, structural remodeling of the membrane lipid is required. This may occur when cells become activated, either by an infectious or non-infectious inflammatory stimulus or following cell damage or apoptosis and may involve the activity of the enzyme phospholipase A2 (6). Upon interaction with the exposed PC groups, pCRP begins to change structure first into an intermediate swollen pentameric form designated pCRP* (or mCRP_m_), then into the fully dissociated, less soluble and antigenically distinct monomeric, modified form, referred to as mCRP (7, 8). Experimental studies have shown that the biological effects of CRP are dependent on its structural conformation, demonstrating strong pro-inflammatory properties of mCRP, whereas pCRP appears to exhibit mainly weak anti-inflammatory activities (9, 10). *In vitro* studies directly comparing the biological effects of the two isoforms, have shown that mCRP has approximately 10-100-fold more potent inflammatory capacity than its precursor molecule pCRP (11).

Notably, once formed, mCRP deposits within tissues due to its low aqueous solubility where it may interact directly with various cells and components of the microenvironment (3, 11). Specifically, it has been shown that mCRP can engage with both epithelial and endothelial cells, platelets, and various immune cells such as macrophages and neutrophils (9, 12, 13). Additionally, mCRP can interact directly with components of the extracellular matrix as well as fibroblasts, which are major constituents of the tumor stroma (3). At the molecular level, data have shown that mCRP preferentially binds to cholesterol rich lipid rafts that are important microdomains of plasma membranes involved in a wide range of cellular processes including signal transduction (9, 14). Following membrane insertion, mCRP can stimulate intracellular signaling including activation of pro-inflammatory pathways such as those involving the pivotal transcription factor NF-κB and its downstream mediators (3).

While most research on the different isoforms of CRP has been carried out in cardiovascular and neurodegenerative disorders, as well as some autoimmune diseases, little is known about their role in cancer (11, 13, 15–18). In line with our previous work (19), focusing on why cancer patients with elevated blood CRP levels have inferior outcomes, the hypothesis evolved that the potent monomeric/modified form of CRP may play a direct pro-inflammatory role within the TME of systemically inflamed cancer patients. First, by localizing the inflammatory response as circulating pCRP binds to exposed PC molecules expressed by cells that have been activated due to the inflammatory TME, leading to *in-situ* dissociation of pCRP into the pro-inflammatory monomeric isoform. Secondly, as mCRP accumulates within the tumor, a process which is considered perpetual and non-resolving, owing to the chronic nature of systemic inflammation, mCRP may play a direct and active role through the recruitment and activation of inflammatory cells and components of the TME, potentially fueling and shaping the local inflammatory response, and ultimately promote tumor progression.

In order to explore whether there is a role for mCRP in systemically inflamed cancer patients, the aim of this proof-of-concept study was to identify and map the pattern of mCRP distribution in a previously immunologically well-defined cohort of colon cancer (CC) patients. Using complementary strategies including immunohistochemistry (IHC)-based colocalization imaging techniques, we were able to elucidate potential functional roles of mCRP in the microenvironment of CC tissue.

## Materials and methods

### Patients and tissue samples

Formalin-fixed, paraffin-embedded (FFPE) tissue samples were retrospectively obtained from 43 stage II and III CC patients, including 20 patients with circulating CRP of 0-1 mg/L (CRP-low patients) and 23 patients with CRP >30 mg/L (CRP-high patients), undergoing resection for their primary tumors at Sørlandet Hospital, Norway, between 2005 and 2015. Clinical information and follow-up data were obtained from a local colorectal cancer database as described previously (19). Characteristics of CRP-high and CRP-low patients are detailed in **Table 1**.

**Table 1:** Clinical characteristics of colon cancer patients according to the level of circulating CRP.

Characteristic	CRP 0-1, N = 20^1^	CRP≥30, N = 23^1^	p-value^2^
Age	67 (60, 71)	78 (71,86)	0.003
Sex			0.70
Female	11 (55%)	14 (61%)	
Male	9 (45%)	9 (39%)	
Stage			<0.001
II	0 (0%)	10 (43%)	
III	20 (100%)	13 (57%)	
Tumor site			0.77
Left	4 (20%)	3 (13%)	
Right	10 (50%)	14 (61%)	
Sigmoid	6 (30%)	6 (26%)	
Adjuvant chemotherapy			<0.001
None	3 (15%)	19 (83%)	
Only 5-FU based	6 (30%)	3 (13%)	
Platinum doublet	11 (55%)	1 (4.3%)	
MMR-Status			0.002
MSS	20 (100%)	14 (61%)	
MSI	0 (0%)	9 (39%)	
Survival status			0.010
Alive	15 (75%)	7 (30%)	
Dead	4 (20%)	9 (39%)	
Recurrence	1 (5.0%)	7 (30%)	
Follow-up (years)	9.3 (8.7, 10.9)	8.8 (5.2, 11.3)	0.58

^1^Median (IQR); n (%).

^2^Wilcoxon rank sum test; Pearson’s Chi-squared test; Fisher’s exact test.

Serum CRP values were determined using a standardized immunoturbidimetric assay, which previously has shown specificity for pCRP without interference with mCRP (20), performed on blood samples taken within 14 days (at the day closest to the resection) prior to the operation in order to reflect a state of chronic inflammation. Exclusion criteria were clinical evidence of infection, use of antibiotics or immunosuppressive drugs within 4 weeks prior to the operation or a history of chronic inflammatory disease including autoimmune disorders.

The study was approved by the Norwegian Regional Ethics Committee.

### Immunohistochemistry and double immunofluorescence

Whole slides from FFPE tumor blocks were immunohistochemically stained with a conformation-specific mCRP monoclonal antibody (mCRP-mAb 9C9), which has been fully characterized previously demonstrating specificity for mCRP and not pCRP (21, 22). FFPE sections were cut at 3 μm, mounted on Superfrost Plus slides (Thermo Fisher Scientific, Waltham, MA), dried for 1 hour at 60°C, and prepared for IHC staining using standard kits from Benchmark Ultra (Ventana, Roche Diagnostics International AG, Basel, Switzerland) for deparaffinization, rehydration, antigen retrieval, and endogenous peroxidase blocking. Next, sections were incubated with the primary antibody (mCRP mAb 9C9 at dilution 1:100) for 30 minutes followed by DAB (3, 3’-diaminobenzidine) substrate chromogen solution for antigen visualization. Negative controls were performed by replacing the primary antibody with antibody diluent (Agilent S2022; DAKO), but otherwise prepared similarly. All sections were counterstained with hematoxylin and mounted before they were scanned at ×20 magnification using NanoZoomer 2.0 HT (Hamamatsu Phototonics KK, Hamamatsu City, Japan).

To map the pattern of mCRP distribution and explore possible colocalization with immune, endothelial and tumor markers, double stainings with chromogenic IHC and IF were performed on tumor slides from selected patients with elevated circulating CRP and pronounced mCRP expression as evaluated by the mCRP single staining. Antibodies against the following markers were applied in addition to anti-mCRP: CD34 for endothelial cells, CD68 for macrophages, CD66b for neutrophils and pan-cytokeratin (pan-CK) as tumor marker. All antibodies were commercially available except for mCRP-mAb 9C9. Origin and incubation times for the applied antibodies are listed in **Supplementary Table S1**.

All double stainings were performed after antigen retrieval as described above. For double IHC, FFPE sections were incubated sequentially, first, with mCRP-mAb at dilution 1:100 for 30 minutes followed by chromogenic DAB staining. The slides were then incubated with the appropriate second primary antibody as listed above at the time indicated for each antibody applying Ultra-view fast red as chromogenic dye. Finally, slides were counterstained with hematoxylin, mounted and scanned at ×20 magnification using NanoZoomer 2.0 HT (Hamamatsu, Japan).

Double IF was performed, using the tyramide signal amplification strategy on the Discovery Ultra Autostainer (Ventana Medical systems) applying two different fluorophores in a sequential manner for visualization of the respective antigens. First, tissue sections were incubated with mCRP-mAb (dilution 1:10) for 30 min, using rhodamine as fluorescent dye, followed by incubation with the appropriate second primary antibody (as listed above) using DCC (N′-dicyclohexylcarbodiimide) as the selected fluorophore. Stained slides were mounted with Vectashield Antifade Mounting Medium, which included DAPI as nuclear counterstain, whereafter they were stored overnight at 4°C, protected from light. Mounted slides were scanned at x 20 using NanoZoomer S60 (Hamamatsu, Japan).

### Digital image analysis

Image analysis was performed using Visiopharm Integrator System software version 2019.02 (VIS; Visiopharm A/S, Hørsholm, Denmark).

**Figure 1:**
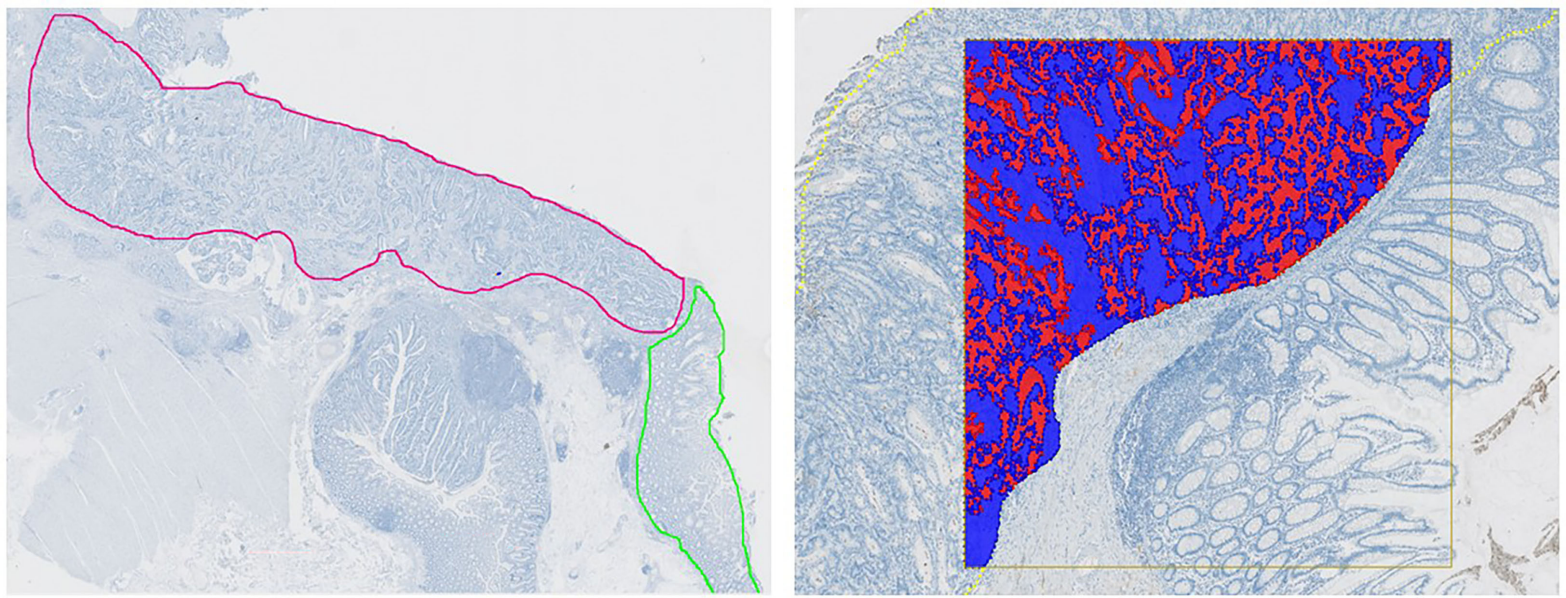
Automated Image Analysis Workflow. Left: Whole slide image with annotated tumor regions. Tumor in red and adjacent normal colon mucosa in green. Right: An AI-based algorithm was developed for analyzing the pattern of mCRP distribution and accurately segment tumor epithelium (red) and tumor stroma (blue).

Regions of interest (ROIs) were defined by a trained pathologist. The tumor was outlined as one region encompassing the invasive margin and tumor center. On slides where normal colon mucosa was present (11 out of 43), this was annotated as a separate ROI. Two AI-based algorithms were utilized for the segmentation and annotation of either tumor epithelium or normal colon mucosa in addition to their surrounding stromal tissue, as outlined in **Figure 1**. Training of the algorithms included a representative set of whole slide images (WSI) where stromal tissue, unstained background, and either tumor epithelium or normal colon mucosa were manually annotated at pixel-level. Using input images of 512 x 512 pixels, U-nets as presented by Ronneberger et al. were trained in VIS’s Author AI (23). Learning rates based on Adam Optimization were set at 1 × 10^−5^, and data augmentation was utilized (24).

In the designated regions outlined by the AI applications, mCRP was identified by thresholding of the brown staining color (DAB), which was highlighted by a color deconvolution step. Post-processing algorithms included morphological operations and changes by area or surrounding. All results of the image analyses were manually reviewed to ensure that areas with mucin, tissue folds, and other technical artefacts were excluded from the analysis.

mCRP was quantified as area proportions defined as: area of positive mCRP staining divided by the total area of the given ROI. Since the area of mCRP was small compared to the total area of the tumor, proportions were multiplied with 1000 and given per mile instead of percentages. Area proportions of mCRP were calculated both as a combined score of total mCRP within the whole tumor as well as separately for the tumor epithelium and tumor stroma, respectively. Finally, mCRP was evaluated within the region of normal colon mucosa, scoring epithelium and stroma combined, on applicable slides.

Double IHC and IF stainings were evaluated and interpreted manually by visual examination only, using NDP. View 2.0 (Hamamatsu).

### Immune phenotypes and microsatellite instability analysis

Immune cell densities (CD8+ T-cells, CD4+ T-cells, Foxp3+ T-cells, CD20+ B-cells, CD66b+ neutrophiles, CD68+ macrophages) assessed within the same tumor regions were captured from a series of multiplexed IHC (mIHC) performed in a previous study (19). However, due to technical issues with the mIHC, 7 patients did not have corresponding immunological profiles and had to be excluded from the mCRP-immune cell correlation analyses.

Mismatch repair (MMR) status was determined by an experienced pathologist through IHC evaluation of the DNA mismatch repair proteins MHL1, MSH2, MSH6, and PMS2. Tumors that were negative in one or more of the four stainings, or inconsistent with IHC, were verified with the Idylla MSI test (Biocartis) as described previously (25). Accordingly, patients were classified as either microsatellite stable (MSS) or instable (MSI).

### Statistical analysis

The distribution of mCRP was assessed as mCRP proportions, as specified above. The median mCRP proportion within groups were calculated and compared using the median test. Differences in patient characteristics were evaluated using Fisher´s exact test and the two-sample t-test with unequal variance. The correlation between mCRP and circulating CRP was assessed using Spearman correlation analysis. Associations between mCRP and the immune markers obtained from mIHC were analyzed using Spearman correlations and heatmaps were generated. The Aalen-Johansen method was applied to estimate the risk of CC death or recurrence and compared between CRP-high and CRP-low patients using the log-rank test. For identification of the most optimal threshold/cutoff value for tumor mCRP expression used in the analysis of the prognostic impact of mCRP, a receiver operating characteristics (ROC) curve was computed. Due to competing risks (death of colon cancer and death of other causes) varying at different time points, the ROC-curve was calculated at the time of median follow-up using the quantified level of mCRP tumor expression for all patients. The optimal mCRP cutoff value was defined as the point on the ROC curve with sensitivity and specificity closest to 100%, which corresponded graphically to the point on the curve with the minimum distance to the upper left corner. The cumulative risk curves for CC death or recurrence are shown for patients with mCRP tumor expression below and above the optimal cutoff value. P<0.05 was considered statistically significant for all analyses. R software version 4.2 was used for statistical calculations.

## Results

### mCRP is expressed predominantly by tumors from systemically inflamed patients and is exclusively present within tumor tissue and not adjacent normal colon mucosa

**Figure 2:**
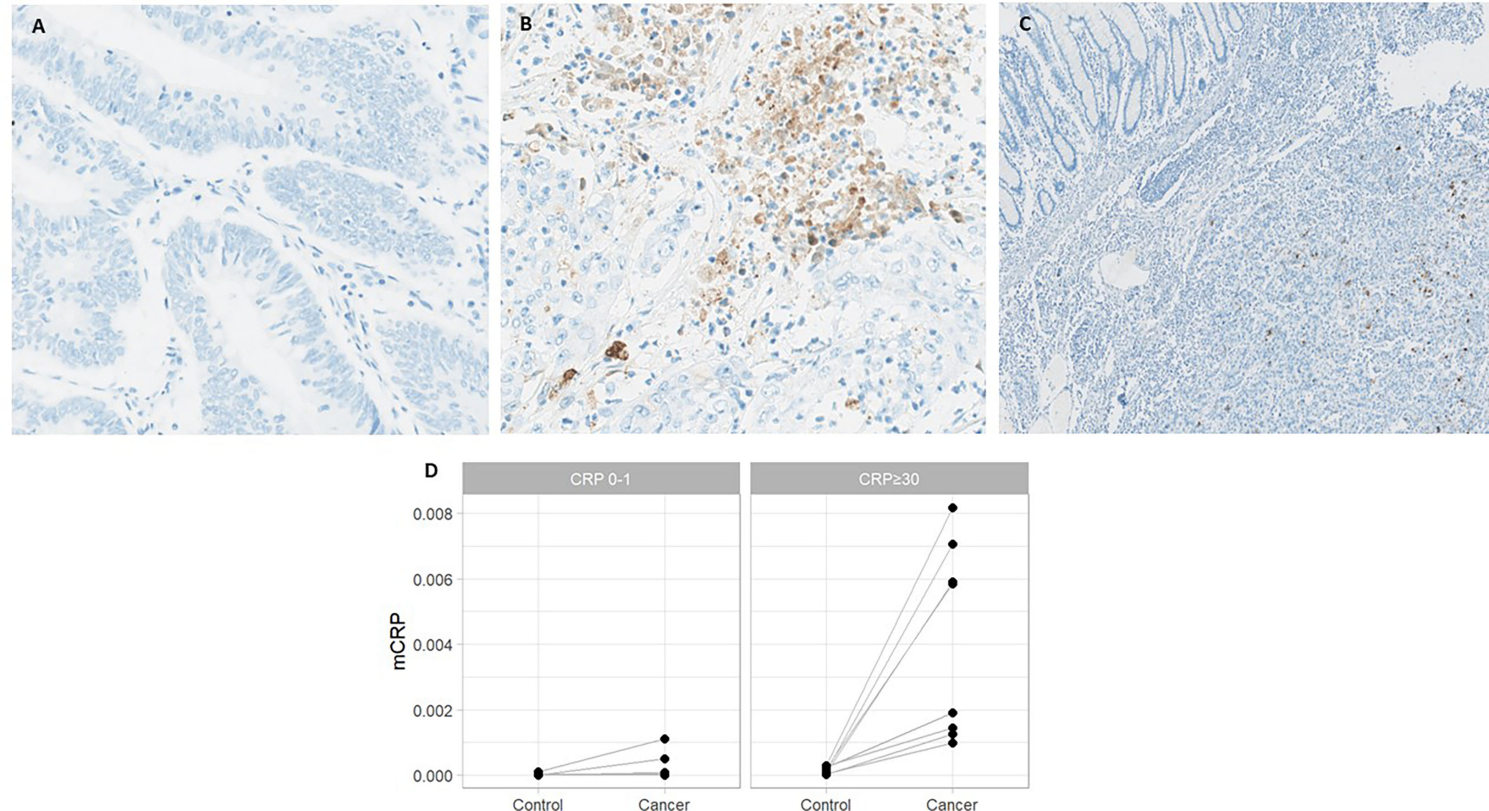
mCRP expression in systemically inflamed and non-inflamed colon cancer patients and adjacent normal colon mucosa. Representative images from patients with **(A)** normal and **(B)** elevated circulating CRP. **(C)** Normal colon mucosa adjacent to the tumor with no mCRP expression. **(D)** Quantified mCRP (proportion of area with positive mCRP staining) assessed within the tumor and adjacent normal colon mucosa (control) in CRP-high and CRP-low patients.

As depicted in **Figure 2**, mCRP was abundantly present in tumors from systemically inflamed CC patients whereas non-inflamed patients exhibited only modest mCRP positivity (median mCRP per area 5.07‰ (95%CI, 1.32-6.85) vs. 0.02‰ (95%CI, 0.01-0.04) p<0.001). Correspondingly, tissue-expressed mCRP correlated strongly with circulating CRP (Spearman correlation 0.81 (95%CI, 0.67-0.89), p<0.001). Further analysis of the pattern of mCRP expression demonstrated that MSI positive tumors exhibited significantly more mCRP compared with CRP-high MSS and CRP-low MSS patients, respectively (data shown in **Table 2**). Furthermore, AI-based image analysis discriminating between tumor epithelium and tumor stroma, showed significantly more mCRP expression in the stromal compartment as compared to the tumor epithelium. Notably, mCRP was detected exclusively within the tumor area whereas adjacent normal colon mucosa showed no mCRP expression (representative image shown in **Figure 2C**).

**Table 2:** mCRP distribution in colon cancer patients stratified for serum CRP and MSI-status.

	n	mCRP stroma	mCRP tumor	P-value
All (per mille), Median (CI)	43	0.70 (0.08-4.33)	0.08 (0.01-0.48)	<0.001
CRP 0-1 (per mille), Median (CI)	20	0.02 (0.01-0.07)	0.00 (0.00-0.01)	<0.001
CRP≥30, MSS (per mille), Median (CI)	14	5.45 (1.79-8.01)	0.33 (0.12-2.87)	<0.001
CRP≥30, MSI (per mille), Median (CI)	9	(3.45-131.76)	2.52 (0.80-13.53)	0.027

Quantification of tissue-associated mCRP expression estimated by IHC.

### Prognostic impact of the CRP isoforms

**Figure 3:**
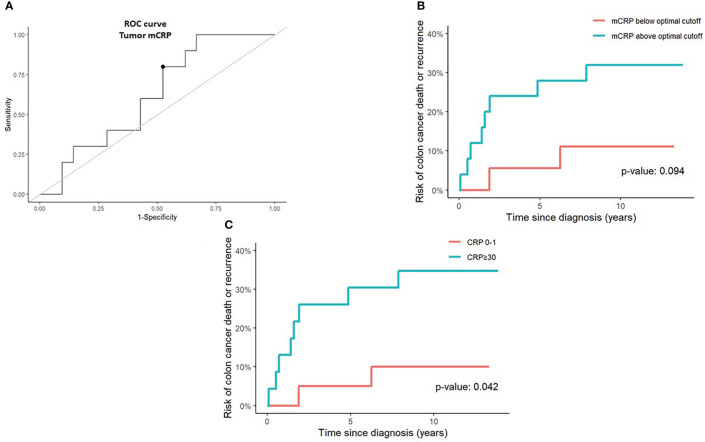
Prognostic value of tumor mCRP expression and serum CRP in colon cancer patients. **(A)** A receiver operating characteristics (ROC) curve was calculated to determine the optimal tumor mCRP cutoff value (marked by a bullet) defined as the point on the curve with sensitivity and specificity closest to 100%, corresponding graphically to the point with the minimum distance to the upper left corner **(B)** Risk of colon cancer death or recurrence above and below the optimal tumor mCRP cutoff value identified from the ROC curve. **(C)** Risk of colon cancer death or recurrence in CRP-high (serum CRP >30 mg/L) and CRP-low (serum CRP 0-1 mg/L) patients. The optimal mCRP cutoff value was defined.

Given the known prognostic role of systemic inflammation and the strong correlation between tissue-bound mCRP and circulating serum CRP, we sought to evaluate whether mCRP had an independent impact on survival outcomes within our cohort. As shown in **Figure 3**, patients with tumors exhibiting mCRP density above the ROC-curve identified cutoff value of tumor mCRP expression tended to perform poorer in terms of increased risk of CC death or recurrence compared with patients that had tumors with mCRP density below the optimal mCRP cutoff value, although this did not reach statistical significance. Nonetheless, elevated serum CRP was confirmed to be predictive of compromised survival and increased risk of recurrence within our cohort (**Figure 3C**).

### mCRP colocalizes with neutrophils and endothelial cells in the TME

**Figure 4:**
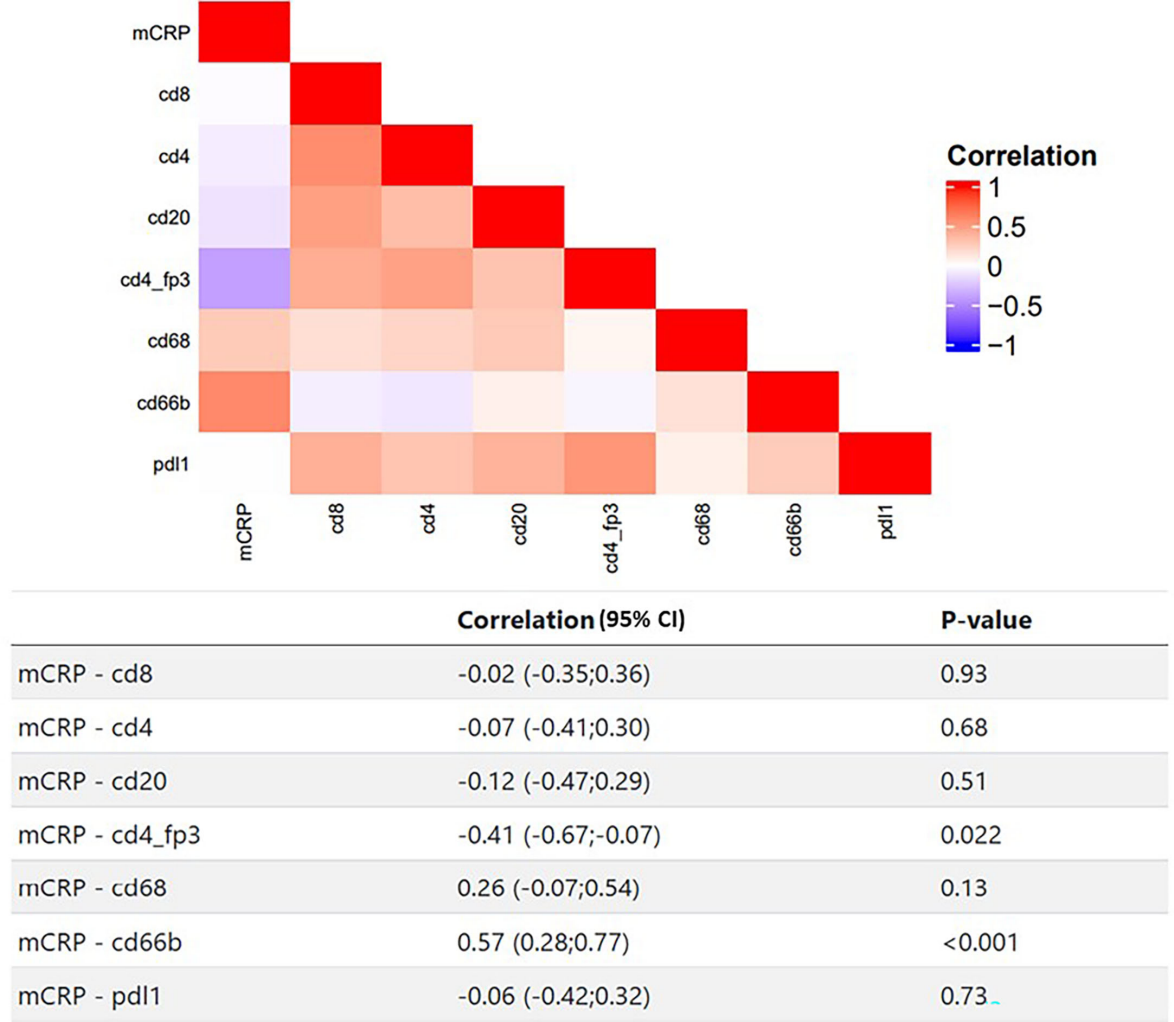
Correlating mCRP and selected adaptive and innate immune markers in colon cancer patients. Heatmap and corresponding table of Spearman correlations between mCRP and individual immune markers. Red color indicates positive correlation, blue indicates negative correlation, white indicates no correlation.

**Figure 5:**
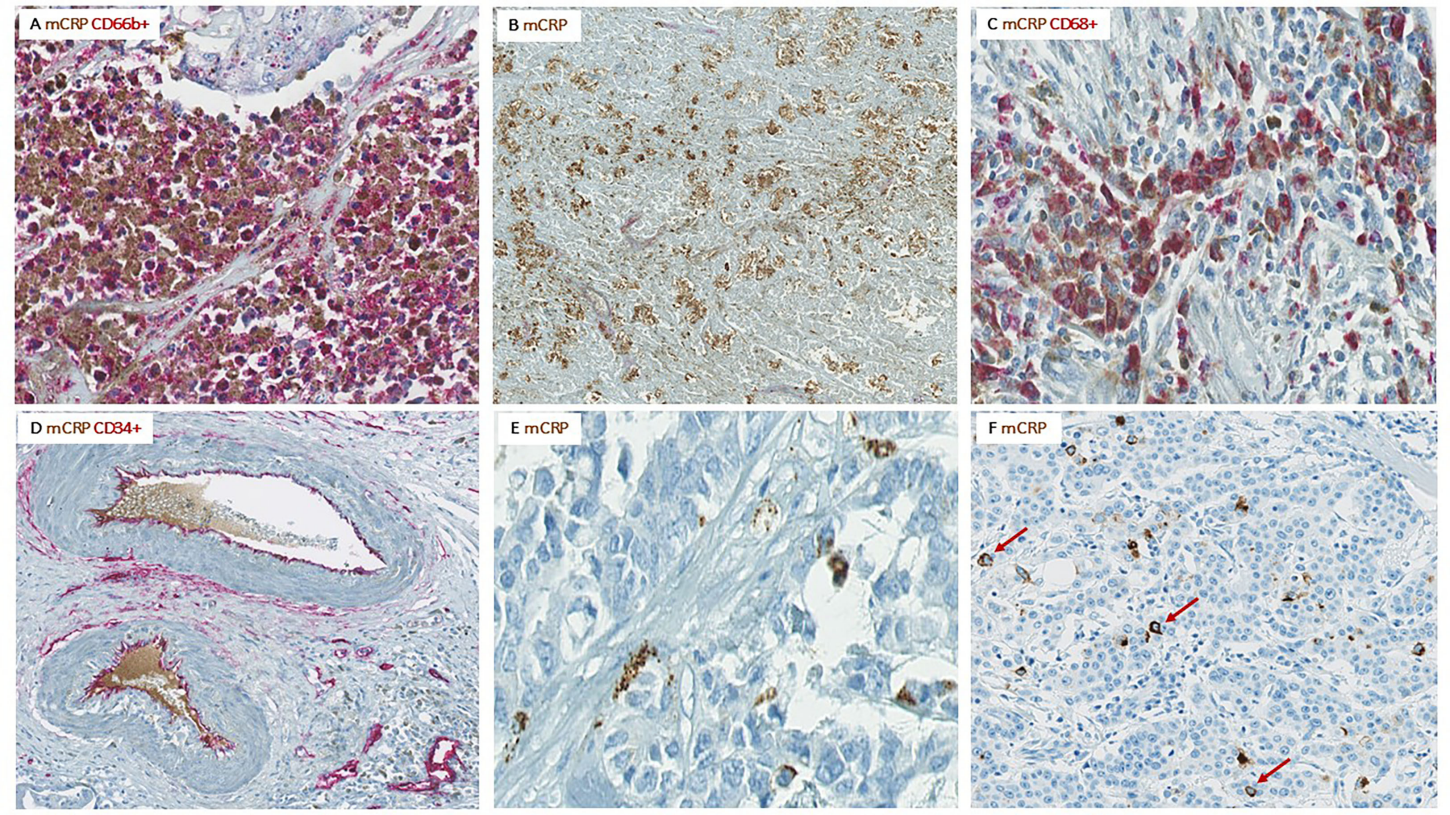
Colocalization of mCRP with various components of the TME. Representative images from CC patients with elevated serum CRP and pronounced mCRP tumor expression. **(A)** Highly neutrophil infiltrated tumor area with strong mCRP expression. **(B)** Necrotic area within a tumor with high mCRP expression. **(C)** Colocalization of mCRP and macrophages. **(D)** Colocalization of mCRP and endothelial cells lining intratumoral vessels as well as some mCRP within the vessel lumen. **(E)** mCRP scattered diffusely as small granules within the connective tissue of the tumor stroma. **(F)** Tumor cell nuclei surrounded by mCRP (marked by arrows).

To elucidate potential functional roles of mCRP in the TME, we took a stepwise approach. First, by performing a correlation analysis of the quantified mCRP IHC results with the immune profiles obtained previously on the same patients and tumor areas, followed by double IHC and IF for mCRP and selected immune and endothelial markers. As shown in **Figure 4** the most evident association was with the neutrophils, showing a highly significant correlation between mCRP and cd66b+ neutrophils (Spearman correlation 0.57, p<0.001). This was supported by double IHC demonstrating strong colocalization of mCRP and areas of neutrophil infiltration (**Figure 5A**). At the sub-cellular level, however, immunofluorescent labeling showed only occasional direct cellular overlap, but confirmed the pattern of close proximity, indicative of an interaction, and to a lesser extent, intracellular uptake of mCRP into the neutrophils.

Moreover, mCRP seemed to coincide with areas of necrosis, with or without neutrophil infiltration, where non-specificity could be ruled out by negative control staining (**Figure 5B**).

Less evident, but still present, was colocalization of mCRP and CD68+ macrophages (**Figure 5C**). However, mCRP-positive macrophages seemed primarily to coincide with highly immune infiltrated areas in general, as the majority of macrophages present more globally dispersed within the tumor tissue showed less mCRP positivity, suggesting that mCRP might be an amplifier of the local inflammatory response.

Based on data from previous studies in cardio- and cerebrovascular diseases, demonstrating a direct interaction between mCRP and endothelial cells, we performed double immune stainings with mCRP and the specific endothelial marker CD34. Notably, mCRP co-localized with endothelial cells lining intratumoral vessels and was present within the lumen of some vessels, suggesting a systemic origin of the monomeric isoform (**Figure 5D**). Additionally, mCRP could be detected within the vessel wall of some mCRP/CD34-positive intratumoral vessels.

Interestingly, in some tumors, mCRP appeared rather scattered around in the tumor stroma, occasionally forming aggregates, but more often globally dispersed as small granules within the connective tissue, suggesting a potential interaction between mCRP and components of the ECM, although this was not directly evaluated by IHC (**Figure 5E**).

### Positive colocalization of mCRP and tumor cells

**Figure 6:**
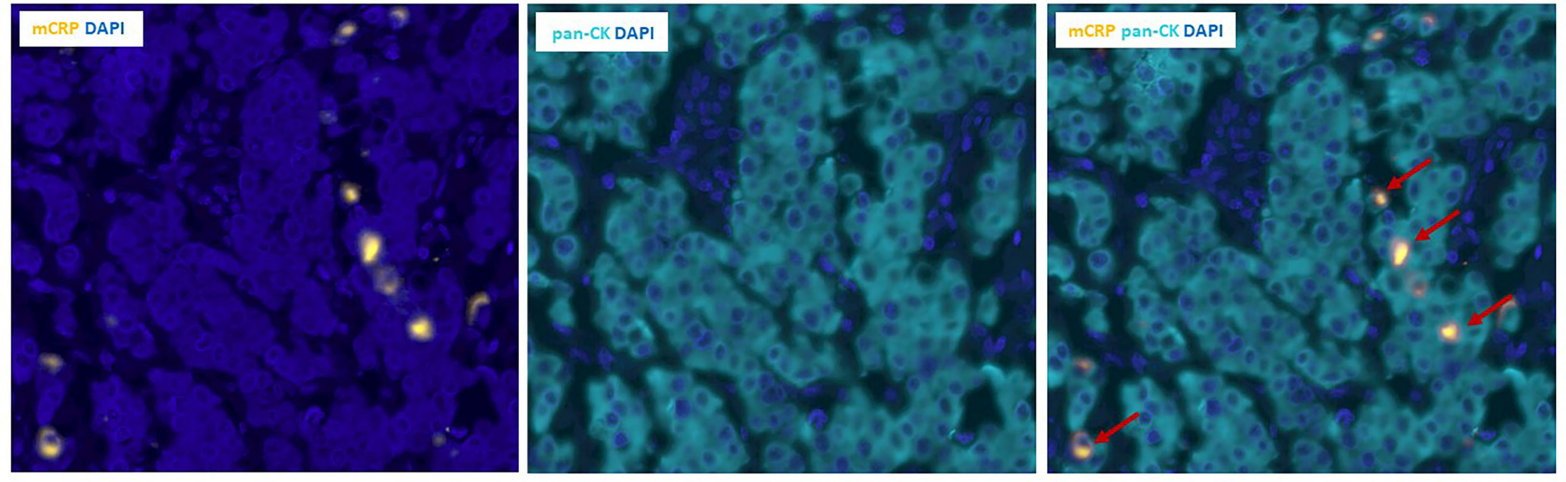
Double immunofluorescence labeling of mCRP and tumor cells in colon cancer tissue. Left and middle panels: Unmixed images showing individual stains of mCRP (yellow) to the left and pan-CK positive tumor cells (teal) in the middle. Right panel: Composite image showing double positive mCRP+/pan-CK+ tumor cells (marked by arrows). DAPI (blue) was used for visualization of nuclei. Pan-CK, Pan-cytokeratin.

Serendipitously, when examining the pattern of mCRP distribution, it became evident that some tumor cells were closely surrounded by mCRP, forming a halo-like coating around individual tumor cell nuclei (**Figure 5F**). To further elucidate this observation, we performed double immune stainings with mCRP and the gastrointestinal specific cytoplasmatic tumor marker pan-cytokeratin. Using double IHC and IF we were able to demonstrate colocalization and evidence of direct overlap of mCRP and tumor cells, indicating close interaction and/or intracellular uptake of mCRP, or potentially, mCRP expression by the tumor itself (representative images shown in **Figure 6**).

## Discussion

In this study we explore the presence of the mCRP isoform and its correlation with innate and adaptive immune cells and serum levels of pCRP in a cohort of stage II and III CC patients. We report that the monomeric form of CRP (mCRP) is present within tumors and that the level of expression correlates strongly with the level of circulating pCRP. Additionally, mCRP expression is associated significantly with tumor infiltrating neutrophils. Importantly, mCRP was expressed exclusively within tumors whereas adjacent normal colon mucosa showed no mCRP positivity.

Persistent elevation of blood CRP levels alongside malignancies is increasingly recognized as an independent predictor of adverse outcomes, both in terms of compromised survival and treatment responses (1, 3, 26). Despite mounting evidence, generated primarily in cardiovascular and neurodegenerative disorders (12, 13, 15, 27, 28), for the existence of different isoforms of CRP with distinct biological properties and direct effects within tissue, this study is the first to apply this emerging concept into the clinical setting of cancer patients. The focus of our previous research has primarily been to understand the biology behind CRP as a biomarker, investigating whether elevated CRP might be a readout of a particular immunological phenotype of the TME. Hence, the idea that CRP itself, in its monomeric, modified form, is present within tumors and might act as a participant in the pathological process has added a new and intriguing layer to this hypothesis and may profoundly change the view on how the local inflammatory response in cancer potentially can be targeted.

Circulating CRP is a pentameric molecule with weak and primarily anti-inflammatory effects through its ability to activate the classical complement pathway, induce phagocytosis and delay apoptosis (10). The much more potent effector function of CRP, however, becomes evident first when pCRP dissociates into the monomeric form exhibiting strong pro-inflammatory properties (12). In cardiovascular disease, it has been shown that activated platelets and endothelial cells, particularly under ischemic conditions, play a pivotal role in the pCRP dissociation process and for the build-up of atherosclerotic plaques (29, 30). Specifically, mCRP and not pCRP, has been detected within atherosclerotic plaques and infarcted myocardium where it co-localizes with oxidized lipoprotein, macrophages and complement factors and is capable of inducing leucocyte migration and adhesion to the endothelium enhancing thrombus formation, excessive inflammation, and ultimately aggravate tissue injury (12, 29). Once formed, *in vitro* studies have shown that mCRP can be inserted into the cell membrane of endothelial cells and activate signaling pathways associated with both angiogenesis and inflammation (14, 29). In line with these findings, we found that mCRP colocalized with endothelial cells lining intratumoral vessels, supporting the hypothesis that endothelial cells, presumably activated by the tumor or the inflammatory microenvironment, is involved in the pCRP-mCRP dissociation process and may contribute to localizing the inflammatory response. Conceivably, newly formed mCRP can then either directly activate the endothelial cells resulting in enhanced leucocyte migration to the tumor, and/or as we demonstrate here, accumulate within the tumor tissue. This occurs particularly in systemically inflamed patients where mCRP may exert its pro-inflammatory effects through direct interaction with different cell types and components of the TME.

To elucidate possible functional roles of mCRP in the microenvironment of our colon tumors, we performed double immune stainings demonstrating prominent colocalization of mCRP and CD66b+ neutrophils. At the sub-cellular level, IF revealed occasional direct cellular overlap, indicating possible uptake of mCRP into the neutrophils, although the predominant pattern was that mCRP coincided with highly neutrophil infiltrated areas, suggesting a close relationship between the two. Given the fundamental role of neutrophil function in acute as well as chronic inflammatory conditions, possible direct effects of CRP on these cells have been of particular interest. Hence, *in vitro* studies have shown that mCRP can delay neutrophil apoptosis and enhance neutrophil adhesion to endothelial cells, which is critical for extravasation of neutrophils into inflamed tissue (31, 32). Additionally, following mCRP stimulation, Kreiss et al. found that neutrophils increased both gene expression and secretion of the pro-inflammatory cytokine IL-8 (33). Intriguingly, growing evidence indicates that IL-8 plays a pivotal role in the TME through the ability to stimulate tumor cell proliferation and promote epithelial-to-mesenchymal transition (EMT), thus facilitating tumor progression and metastasis (34).

We have previously shown that elevated circulating CRP associates with a neutrophil enriched and immunosuppressive TME (19). Together with these findings suggesting direct crosstalk between mCRP and neutrophils, this does not only reinforce a profound role for neutrophils in the microenvironment of tumors but adds new information on why neutrophils, particularly during a chronic inflammatory state, seem to be such potent players favoring a detrimental inflammatory response and subsequently how this potentially can be targeted.

Of note, we also observed that mCRP seemed to coincide with areas of necrosis, with or without neutrophil infiltration, showing a pattern of high mCRP expression within and in the vicinity of necrotic areas. This phenomenon could be related to the notion that mCRP can induce aberrant angiogenesis, which has been shown in infarcted brain tissue, resulting in leaky vessels that compromise sufficient blood supply to the tumor leading to necrosis (35). In cancer biology, necrosis is associated with poor prognosis and treatment resistance and has been linked to an immunosuppressive microenvironment, possibly through the release of damage-associated molecular patterns (DAMPs) from dying cells, which triggers an inflammatory response (36). Hence, the ability of mCRP to induce tumor necrosis could potentially contribute to a hostile and predominant immunosuppressive microenvironment supporting a more aggressive tumor phenotype.

Within this context it should be mentioned that a series of older studies conducted in various experimental, primarily murine, cancer models, using CRP, either in its pentameric form or injecting mCRP directly, found similar correlation with necrosis as demonstrated in the present study (11). Contrary to our hypothesis, though, the addition of CRP to the experimental models associated with tumor regression and anti-metastatic effects. However, within all these experimental set-ups CRP was applied only for a short period of time (weeks) and primarily as boosts with CRP injection on selected days. Hence, such system models would mimic an acute inflammatory response and not the situation during chronic systemic inflammation, which was the case for the patients within our cohort. In cancer patients with persistent elevation of blood CRP levels, the inflammation is proposed to be sustained due to the ongoing inflammatory stimulus from the evolving tumor that potentiates hepatic and potentially, tumor intrinsic CRP production, leading to the “wound that never heals”. Considering the pro-inflammatory effects of mCRP together with the capacity of activated cells to induce pCRP dissociation, persistent pCRP exposure may then result in excessive tumor inflammation and tissue damage ultimately facilitating tumor growth and exacerbation of the disease.

Previous studies have demonstrated that mCRP can interact with components of the ECM, such as collagen, fibronectin and laminin, which are integral parts of connective tissues playing a crucial role for tissue maintenance and homeostasis (11, 37, 38). In tumors, however, this highly dynamic network becomes dysregulated, and together with other components of the tumor stroma, contributes to a tumor permissive microenvironment. Importantly, low tumor-stroma ratio associates with poor survival and treatment outcome in multiple cancer types (39, 40). In our cohort, we found that mCRP, in addition to the above-described distribution pattern, often was scattered diffusely as small granules embedded within the stroma, unrelated to any particular cell type. Consistent with previous studies delineating the precise ligands for mCRP (5), this morphological pattern could indicate possible crosstalk between mCRP and components of the ECM. Given the putative pro-inflammatory properties of mCRP, such direct interactions could potentially contribute to excessive stromal formation. Apart from enlargement of the tumor, abundant ECM deposition has been linked to increased stromal stiffness, which subsequently can contribute to treatment resistance and favor tumor aggressiveness (40).

Serendipitously, when examining the pattern of mCRP distribution, it became apparent that some tumor cells were decorated by mCRP. Using double immune stainings with pan-cytokeratin as a tumor marker, we found evidence of direct overlap indicating close interaction and/or mCRP expression by tumor cells. Whether the positive mCRP/tumor staining depicts direct uptake of mCRP into tumor cells or represents an intrinsic feature that the evolving tumor acquires to support its own growth and formation of a tumor permissive microenvironment, remains elusive and should be expanded on in further studies.

Indeed, studies have shown that although the liver is the main source of CRP, extrahepatic production do exist (10, 41, 42). Specifically, macrophages, endothelial cells, smooth muscle cells as well as adipocytes and lymphocytes have been reported to synthesize CRP (10). Hence, we cannot rule out that the observed intratumoral mCRP is produced locally by inflammatory and/or tumor cells. The strong correlation with circulating serum CRP, however, indicates that the primary source of tissue-associated mCRP in our tumors was from systemic pCRP. Nonetheless, regardless of origin, given the evidence described above, persistent presence of mCRP within the tumor, which is considered an ongoing, non-resolving state due to the chronic nature of tumor-associated systemic inflammation, may potentially play a direct and active role in aggravating the localized inflammatory response. Notably, the versatile binding capacity of mCRP to a number of different cellular and non-cellular ligands, may potentially translate into multiple effects within the TME through its direct interaction with diverse targets that most likely will impact the evolving tumor.

This study has several limitations. Above all, it is a proof-of-concept study primarily performed for testing hypotheses and exploring a rather new and, in our opinion, underappreciated concept in clinical oncology, thus limiting the sample size. Hence, our findings need to be verified and further explored in larger studies, which we are currently conducting. Next, we used FFPE tissue and IHC to elucidate possible functional roles of mCRP within tumors. While this methodological strategy provides high morphological precision regarding localization of the applied markers, the ability to evaluate direct functionality is, however, limited. This aspect should therefore be addressed in other kind of experiments, preferentially using fresh tissue. Finally, our tumor samples, although whole slides, only represent a snapshot of the immunological process, and do not mirror the long-term conditions and temporal dynamics. Hence, serial biopsies will be valuable to further dissect and evaluate how mCRP affects the immune response over time and impacts tumor evolution.

Taken together, we provide evidence for the existence of the monomeric form of CRP in CC being expressed exclusively within tumor tissue, primarily in systemically inflamed patients. mCRP expression colocalized with neutrophils and endothelial cells as well as areas of necrosis indicating a direct role in the microenvironment of tumors. In line with findings from studies conducted in other diseases, we suggest mCRP as a potential tissue-associated player with capability of actively shaping and fueling the local tumor immune response, presumably by creating a more tumor permissive environment and negatively affect patient outcome. These findings, if verified in further studies, puts CRP in a new perspective, acting not only as a biomarker of unfavorable prognosis and outcomes in cancer, but also as an active mediator with direct effects within tumors, and opens a new and intriguing approach for targeting the TME.

## Data availability statement

The original contributions presented in the study are included in the article/**Supplementary Material**. Further inquiries can be directed to the corresponding author.

## Ethics statement

The studies involving human participants were reviewed and approved by Norwegian Regional Ethics Committee. Written informed consent for participation was not required for this study in accordance with the national legislation and the institutional requirements.

## Author contributions

AK contributed to data collection, analysis, and interpretation, and drafted the manuscript. JG, KC, and HZ prepared human tissue and performed lab work. PN performed digital image analysis and data interpretation. AF, IR, and LP provided materials, contributed to data interpretation and discussion of content. ET conducted statistical analyses and data curation. TS contributed to data interpretation, methods and discussion of content. CK participated in data interpretation, discussion of content and conceptual framework. All authors contributed to the article and approved the submitted version.
