# Supplementary material for: Fueling the flames of colon cancer – does CRP play a direct pro-inflammatory role?
Source: Front Immunol. 2023 Mar 17;14:1170443. doi: 10.3389/fimmu.2023.1170443 (PMC10065292; doi:10.3389/fimmu.2023.1170443)
Supplement: Supplementary file 1 [file Table_1.docx]

**Table S1 Origin and incubation times for the antibodies used for IHC double staining**

| **Target** | **Clone** | **Vendor** | **Specie** | **Dilution** | **Incubation** |
| --- | --- | --- | --- | --- | --- |
| CD66b | G10F5 | Beckman Coulter | mouse | 1:200 | 40min |
| CD68 | PG-M1 | Dako | mouse | 1:100 | 32min |
| CD34 | Qbend10 | Ventana | mouse | 1:100 | 60min |
| pan-CK | Ae1/3 | Dako | mouse | 1:100 | 32min |
| mCRP-mAb | 9C9 | Prof. Potempa | mouse | 1:100^*^/1:10^**^ | 32min |

*single and double chromogenic IHC **double immunofluorescense
